# Zone 2 hybrid thoracic endovascular aortic repair: Is it a good option for all types of thoracic aortic disease?

**DOI:** 10.1186/s13019-022-01798-7

**Published:** 2022-03-25

**Authors:** Bongyeon Sohn, Jae Hang Lee, Joon Chul Jung, Hyoung Woo Chang, Dong Jung Kim, Jun Sung Kim, Cheong Lim, Kay-Hyun Park

**Affiliations:** Department of Thoracic and Cardiovascular Surgery, Seoul National University College of Medicine, Seoul National University Bundang Hospital, Bundang-gu, Seongnam-si, Gyeonggi-do 13620 Republic of Korea

**Keywords:** Aortic aneurysm, Aortic dissection, Hybrid, Stent, Endovascular repair

## Abstract

**Background:**

Zone 2 thoracic endovascular aortic repair (TEVAR) is performed for the treatment of various thoracic aortic diseases involving the left subclavian artery. This study aimed to analyze the late clinical outcomes of zone 2 hybrid TEVAR according to the various indications.

**Methods:**

A total of 48 patients who underwent zone 2 TEVAR at our institution between December, 2010 and July, 2020 were enrolled. The indications were aortic aneurysm (AA, n = 15), acute type B aortic dissection (AD, n = 14), penetrating aortic ulcer (PAU, n = 8), traumatic aortic injury (TAI, n = 8), and others (n = 3). The clinical outcomes including early complications and mid-term aortic measurements were retrospectively reviewed.

**Results:**

The technical success rate was 100% and in-hospital mortality occurred in one patient. The early postoperative complications included stroke (n = 1), transient spinal cord ischemia (n = 1), neck wound hematoma (n = 1), and left phrenic or vagus nerve injury (n = 9). In patients with AD, positive remodeling was observed in ten patients (76.9%) (false lumen regression in the entire or thoracic aorta [n = 9], false lumen thrombosis in the thoracic aorta [n = 1]). However, in patients with AA, increased aneurysm was found in six patients (40%). Persistent aneurysmal growth was found in patients with a maximal aortic diameter of > 60 mm on initial imaging (4/6, 50%). No aortic expansion was observed in those with TAI or PAU. Endoleak was noted in five patients (10.4%), and among them, aortic reintervention was required only in patients with large AAs.

**Conclusions:**

Zone 2 hybrid TEVAR was associated with an acceptable early complication rate and provided acceptable mid-term aortic results for patients with AD, PAU, and TAI. However, patients with large AAs were at increased risk of aortic reintervention. In cases of large AA, clinicians should carefully consider whether zone 2 hybrid TEVAR or open surgical repair will be more effective for the patient.

## Background

Thoracic endovascular aortic repair (TEVAR) is widely applied and accepted as a first-line method of treatment for various thoracic aortic diseases [1, 2]. It is known that 40–50% of patients undergoing TEVAR require left subclavian artery coverage due to disease extension into the aortic arch [3]. In this case, zone 2 TEVAR is considered to address the insufficient proximal landing zone distal to the left subclavian artery. However, previous studies on zone 2 TEVAR typically focused on strategies for left subclavian artery coverage, and the application of routine revascularization of the left subclavian artery in the hybrid arch procedure is controversial. In addition, the long-term efficacy of zone 2 TEVAR for the various indications not been clarified. Therefore, this study aimed to present our center experience of zone 2 hybrid TEVAR in the past 10 years, and to compare the clinical outcomes of this procedure according to the various types of thoracic aortic disease being treated.

## Material and methods

### Patient population and inclusion criteria

The 48 patients who underwent zone 2 TEVAR at our institution between December 2010 and July 2020 were enrolled in this study. The indications for zone 2 TEVAR were aortic aneurysm (AA, n = 15), acute aortic dissection (AD, n = 14), penetrating aortic ulcer (PAU, n = 8), traumatic aortic injury (TAI, n = 8), and others (n = 3). Others (n = 3) included graft anastomosis site pseudoaneurysm after aortic surgery. Acute type B complicated AD with a large entry tear and saccular or fusiform thoracic aortic aneurysm were the main indications for zone 2 TEVAR. Uncomplicated AD and chronic type B AD are not indications for TEVAR at our institution. The study protocol was reviewed by the institutional review board and was approved as a minimal-risk retrospective study (IRB Approval Number: B-2101/661–105) that did not require individual consent. The baseline characteristics are presented in Table 1.

**Table 1:** Baseline characteristics

Variables	Mean ± SD or n (%)
Age, y	62.0 ± 15.3
Male, n (%)	35 (66.0)
Hypertension, n (%)	32 (60.4)
Dyslipidemia, n (%)	13 (24.5)
Diabetes mellitus, n (%)	8 (15.1)
Cerebrovascular accident history, n (%)	4 (7.5)
Chronic obstructive pulmonary disease, n (%)	7 (13.2)
Peripheral arterial occlusive disease, n (%)	5 (9.4)
Chronic kidney disease, n (%)	2 (3.8)
Coronary arterial occlusive disease, n (%)	8 (15.1)

*SD* Standard deviation

### Surgical procedures and strategy

Both femoral arteries and aortic arch vessels including the left subclavian artery were routinely evaluated preoperatively using computed tomography (CT) angiography. The selection of all devices was at the discretion of the surgeons. Emergency surgery was performed in six cases (12.5%), and hybrid arch repair with concomitant left common carotid artery to left subclavian artery bypass was routinely performed with the exception of these emergency cases. All patients underwent the TEVAR procedure under general anesthesia in a hybrid operating room. Left subclavian artery bypass was performed using woven double velour polyester grafts (Hemashield Platinum, Maquet) 6 mm or 8 mm. To prevent type II endoleaks, proximal left subclavian artery embolization was routinely performed using an Amplatzer Vascular Plug II (AGA Medical Corporation, Plymouth, MN, USA). The length of the proximal landing zone was secured at ≥ 15 mm for all cases considering the manufacturers’ instructions for use. Cerebral oximetry using near‐infra red spectroscopy to assess cerebral oxygenation levels was applied for the patients who underwent left common carotid artery to left subclavian artery bypass surgery. Intraoperative cerebrospinal fluid drainage was performed when necessary.

### Evaluation of clinical outcomes

The data regarding postoperative outcomes and events were acquired by reviewing the medical records or from direct telephone interviews with the patients or their families. All patients were successfully followed-up for a mean period of 3.1 ± 2.5 years. Early mortality was defined as any death within 30 days after surgery or during the same hospitalization period. Immediate postoperative CT was performed within 1 month after the operation. Postoperative follow-up was performed regularly in an outpatient clinic at 3- to 12-month intervals. Annual CT follow-up was performed. Technical success was defined as proper deployment of the graft at the landing zone as preoperatively planned, with total coverage of the lesion.

## Results

### Early outcomes

The technical success rate was 100% and no patient underwent or converted to open surgical aortic repair after the introduction of TEVAR. The operative data are shown in Table 2.

**Table 2:** Operative data

Variables	
*Initial diagnosis*	
Degenerative aneurysm, n (%)	15 (31.3)
Acute aortic dissection, n (%)	14 (29.2)
Penetrating aortic ulcer, n (%)	8 (12.5)
Traumatic aortic injury, n (%)	8 (12.5)
Others, n (%)	3 (6.3)
*Stent grafts*	
Valiant captivia (medtronic), n (%)	28 (58.3)
SEAL (S&G Biotech), n (%)	18 (37.5)
Zenith TX2 (Cook), n (%)	2 (4.2)
LCCA to LSCA bypass, n (%)	43 (89.6)
Emergency, n (%)	6 (12.5)

*LCCA* Left common carotid artery, *LSCA* left subclavian artery

In-hospital mortality occurred in one patient (2.1%). The cause of early death was bowel ischemia and the patient had been preoperatively diagnosed with severe visceral malperfusion due to acute AD. Postoperative stroke was diagnosed in one patient (2.1%); multiple embolic infarction was found on the brain imaging. Transient spinal cord ischemia was occurred in one patient (2.1%) and the patient was discharged without any neurological deficit. Complications related to the left subclavian artery bypass procedure were noted in several patients, including left phrenic nerve palsy (n = 7, 14.6%), left vagus nerve palsy (n = 2, 4.2%), and cervical wound problems (n = 1, 2.1%). Phrenic nerve palsy was diagnosed when left side diaphragm elevation was found on chest X-ray. Vagus nerve palsy was diagnosed by a vocal fold exam for patients presenting with hoarseness. These complications did not influence critical care and hospital stay. All patients were weaned from the mechanical ventilator within the first postoperative day. The weaning process did not require reintubation. No significant difficulties in swallowing function were noted. They were monitored during the outpatient follow-up period; all patients exhibited improvement and are presently symptom-free (Table 3).

**Table 3:** Early outcomes

Variables	
Technical success, n (%)	48 (100)
Mortality, n (%)	1 (2.1)
Stroke, n (%)	1 (2.1)
Transient spinal cord ischemia, n (%)	1 (2.1)
LCCA-LSCA bypass related complications	
Cervical wound hematoma requiring exploration, n (%)	1 (2.1)
Left phrenic nerve palsy, n (%)	7 (14.6)
Left vagus nerve palsy, n (%)	2 (4.2)

*LCCA* Left common carotid artery, *LSCA* left subclavian artery

### Aortic measurements after zone 2 hybrid TEVAR for various thoracic aortic diseases

#### Acute type B AD

**Fig. 1:**
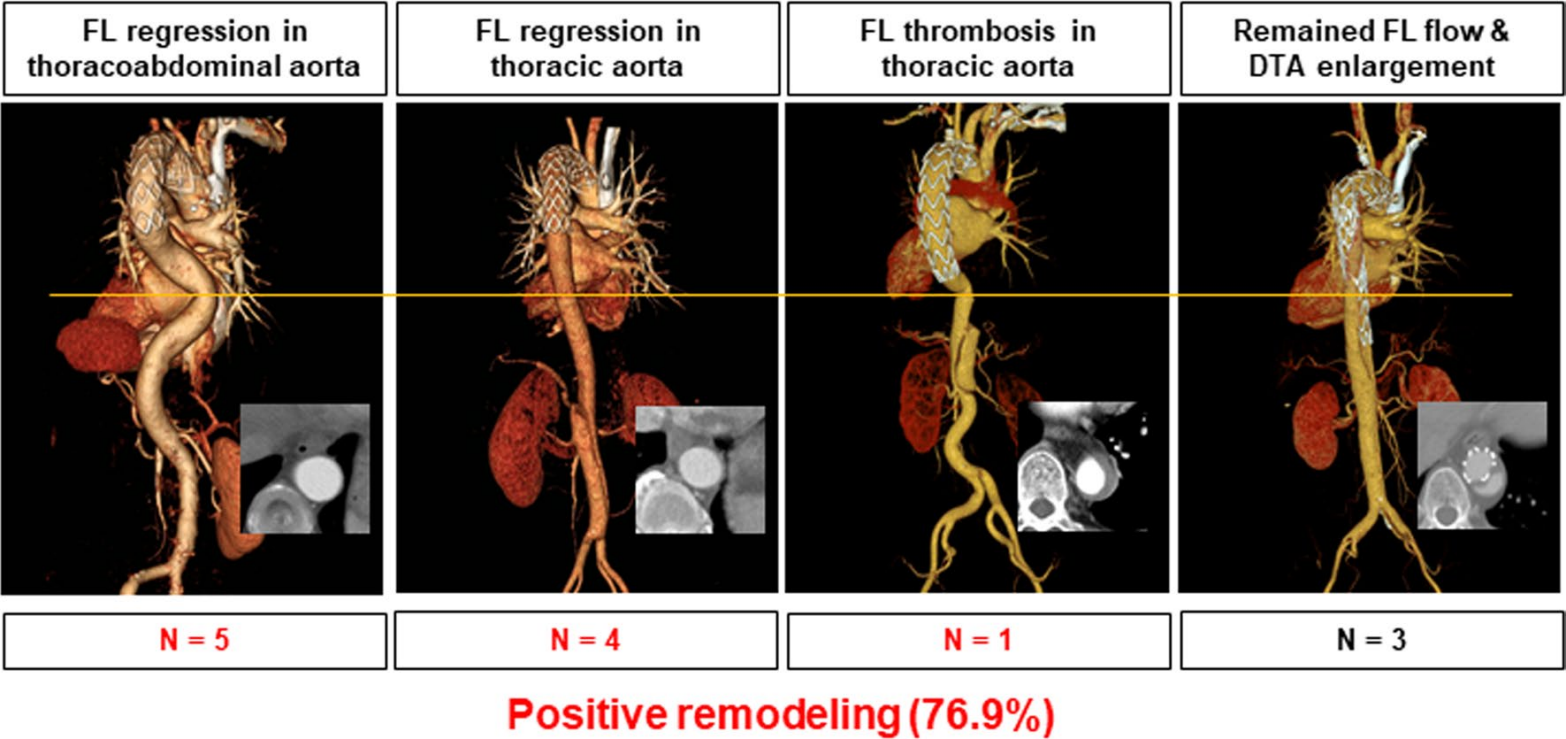
Aortic measurements after zone 2 hybrid thoracic endovascular aortic repair for acute type B aortic dissection. The transverse line on the figure points the distal descending thoracic aorta level. The same level of CT axial images is presented together. False lumen regression over the entire thoracoabdominal aorta was observed in five patients (38.5%), false lumen regression at the thoracic aorta was observed in four patients (30.8%), partial false lumen thrombosis of the thoracic aorta was found in one patient (7.7%), and remaining false lumen flow or enlargement of the descending thoracic aorta was noted in three patients (23.0%). The rate of positive aortic remodeling was 76.9%. FL, false lumen; DTA, descending thoracic aorta

Among the 14 cases of AD, one case of early mortality was excluded from the analysis of the anatomical effects of zone 2 TEVAR. A significant reduction in the false lumen diameter with a corresponding increase in the true lumen diameter after stent-graft placement was observed. Figure 1 shows the postoperative CT angiography and three-dimensional reconstruction images. False lumen regression was defined as remaining false lumen thrombosis with a thickness of < 5 mm. False lumen regression over the entire thoracoabdominal aorta was observed in five patients (38.5%), false lumen regression at the thoracic aorta was observed in four patients (30.8%), partial false lumen thrombosis of the thoracic aorta was found in one patient (7.7%), and remaining false lumen flow or enlargement of the descending thoracic aorta was noted in three patients (23.0%). Thus, the rate of positive aortic remodeling as the expansion of the true lumen and shrinkage or occlusion of the false lumen after zone 2 TEVAR for AD was 77%.

### AA

**Fig. 2:**
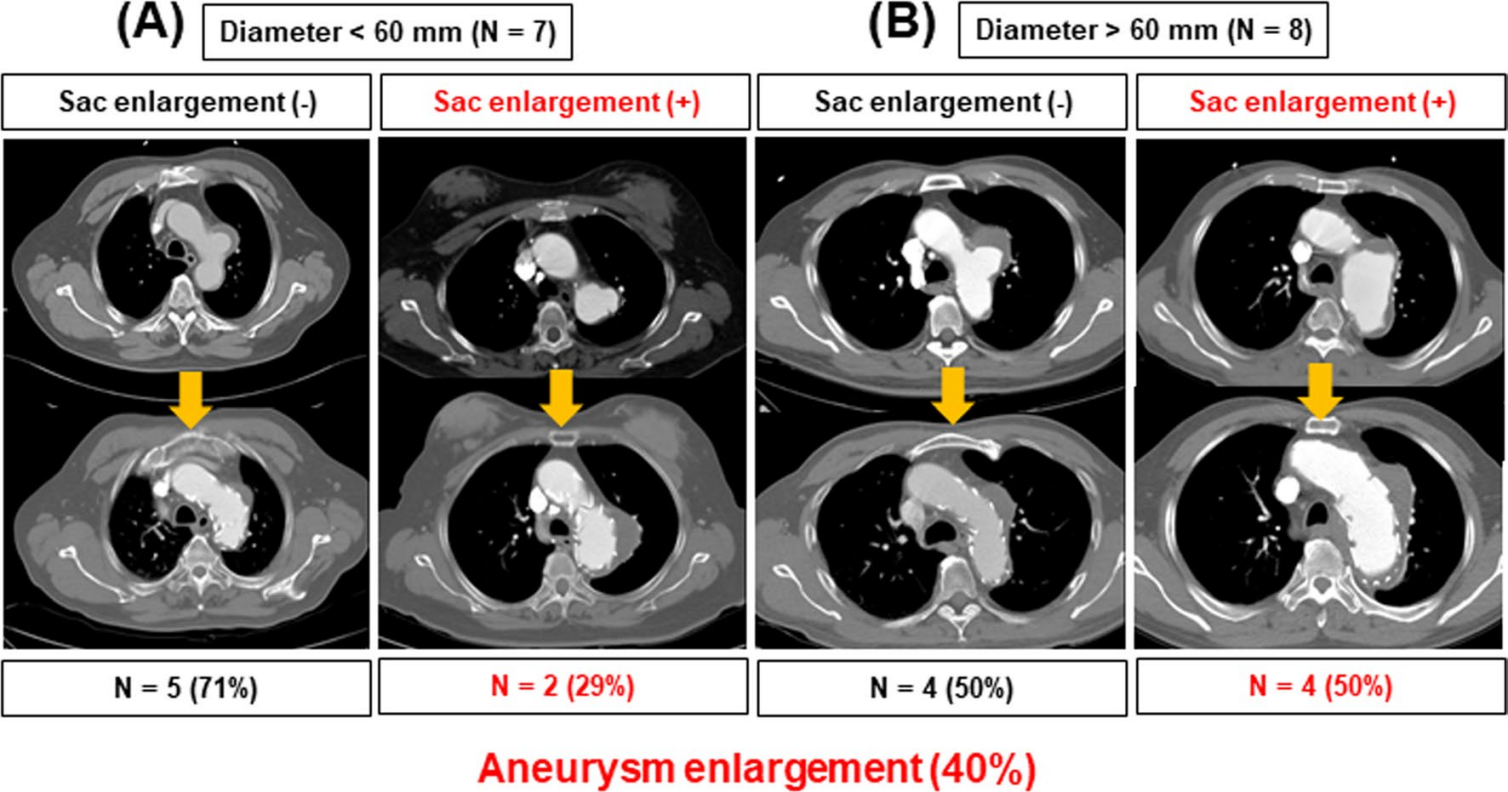
Aortic measurements after zone 2 hybrid thoracic endovascular aortic repair for aortic aneurysm. The 15 patients with aortic aneurysm were divided into two groups according to the initial maximal diameter of the aortic aneurysm to analyze the effect of the aneurysm size: **A** < 60 mm (n = 7) and **B** > 60 mm (n = 8). Among the patients of group (**B**), 50% exhibited aneurysmal enlargement after procedure

The change in aneurysm size after zone 2 TEVAR was evaluated. The 15 patients with AA were divided into two groups according to the initial maximal diameter of the AA to analyze the effect of the aneurysm size: < 60 mm (n = 7) and > 60 mm (n = 8). Among the patients with a maximal aortic diameter of < 60 mm, persistent aneurysmal sac enlargement was observed in two (29%). However, among the patients with a maximal aortic diameter of > 60 mm, four (50%) exhibited aneurysmal enlargement after zone 2 TEVAR. A large AA (diameter > 60 mm) was associated with a higher incidence of aneurysmal enlargement after zone 2 TEVAR (Fig. 2).

#### PAU and TAI

Near resolution of aortic ulcer or aortic injury was found on immediate postoperative CT. AA shrinkage was observed in all patients with PAU (n = 8). The complete exclusion of the primary target lesion was found in all patients with TAI (n = 8), and no aortic expansion was observed on follow-up CT.

### Endoleak and aortic reintervention

Late endoleak after zone 2 TEVAR was confirmed in five patients (10.4%) on follow-up CT. Type Ia endoleak was noted in three patients and type Ib endoleak was observed in two patients. Other types of endoleak were not observed. All patients showed good left common carotid artery to left subclavian artery bypass patency. The patients determined to have type Ia endoleak were all initially diagnosed with AA. Among them, two underwent total arch replacement due to endoleak with persistent aneurysmal growth. The remaining one patient also exhibited an increase in aneurysm size, but did not undergo aortic reintervention due to old age. The two cases of type Ib endoleak occurred in patients with AA and AD. Aortic reintervention was not required in these two patients since aneurysmal growth was not observed. For reasons unrelated to endoleak, one aortic reintervention was implemented. Retrograde type A AD occurred in one patient 3 weeks after TEVAR. The patient was re-hospitalized and underwent total arch replacement. Thus, the aortic reintervention rate after zone 2 hybrid TEVAR was 6.3% (3 of 48 patients, Table 4).

**Table 4:** Aorta-related outcomes of various thoracic aortic diseases

	AD (N = 13)	AA (N = 15)	PAU (N = 8)	TAI (N = 8)
Aortic remodeling				
Positive remodeling, n (%)	10 (76.9)	–	–	–
Failure of remodeling, n (%)	4 (23.1)	–	–	–
Aneurysm enlargement				
Persistent growing, n (%)	–	6 (40.0)	–	–
Shrinkage of aneurysm, n (%)	–	9 (60.0)	8 (100.0)	8 (100.0)
Endoleak				
Type Ia, n (%)	–	3 (20.0)	–	–
Type Ib, n (%)	1 (7.7)	1 (6.7)	–	–
Aortic re-intervention, n (%)				
Open surgical replacement	–	2 (13.3)	1 (12.5)	–
Additional endovascular repair	–	–	–	–

*AD* Aortic dissection; *AA* aortic aneurysm; *PAU* penetrating aortic ulcer; *TAI* traumatic aortic injury. Among the 14 cases of AD, one case of early mortality was excluded from the analysis of the anatomical effects

## Discussion

This study aimed to compare the late clinical outcomes of zone 2 hybrid TEVAR according to the various types of thoracic aortic disease being treated. We yielded three main findings: first, zone 2 TEVAR showed acceptable and durable mid-term outcomes for various thoracic aortic diseases, particularly acute type B complicated AD, PAU, and TAI. Second, the concomitant left carotid artery to left subclavian artery bypass with left subclavian artery embolization procedure during zone 2 TEVAR was performed safely and resulted in lasting bypass graft patency. Third, large AAs with an initial diameter of > 60 mm were associated with higher risks of persistent aneurysmal growth and endoleaks after zone 2 TEVAR.

The indications for hybrid TEVAR with the debranching arch procedure have been gradually extended. This procedure provides an adequate proximal landing zone for TEVAR in patients with aortic disease with aortic arch involvement. However, there are still fatal problems with this procedure, such as retrograde type A AD, stroke, and persistent endoleak in regard to the long-term outcomes. Previous studies have reported higher aortic reintervention rates after TEVAR with the hybrid arch procedure than after open aorta replacement for AA [4, 5]. A recent study investigating the late outcomes of hybrid aortic arch repair for both AD and degenerative AA revealed a high incidence of complications within a median follow-up period of 60 months and recommended life-long surveillance in order to achieve better outcomes [6].

As TEVAR became more common, clinical studies began to focus not only on mortality, but also on aortic remodeling in association with late complications. Our current study found favorable aorta-related results of zone 2 hybrid TEVAR for acute type B AD, PAU, and TAI relative to previous studies. In cases of acute type B AD, the rate of positive aortic remodeling (true lumen re-expansion and thoracic false lumen thrombosis) was as high as 77%. This was slightly lower than the rate of 80–90% reported in previous studies investigating remodeling rates of general TEVAR for AD [7]. However, we exclusively reported the outcomes of zone 2 hybrid TEVAR for acute type B AD with a low number of procedure-related complications and sustainable mid-term aortic remodeling rates. Since the aortic remodeling rate is also associated with the follow-up duration, it is expected to increase after a longer period of observation. Multiple studies of type B AD have demonstrated that TEVAR promotes aortic remodeling as early as 6–12 months postoperatively [8, 9]. Our data support that early aortic remodeling after zone 2 TEVAR is observed even in the immediate postoperative period and can be consistently identified on follow-up CT. Good results were also obtained for PAU and TAI, relative to previous findings. A Chinese group published the 5-year outcomes of TEVAR for symptomatic PAU; they reported two cases of early type II endoleaks, but no aortic expansion was observed and no patients needed reintervention. They also found that PAU typically appeared in the segment extending from the left subclavian artery to the middle descending aorta (24/28, 89%) [10]. Therefore, zone 2 TEVAR is a good option for most patients with PAU. However, despite the good early outcomes for TAI, there are concerns regarding the long-term aorta-related outcome [11]. Secondary aortic interventions rates were high (22%) in a previous study of the efficacy of zone 2 TEVAR for TAI [12]. However, our current study demonstrated sustainable mid-term outcomes of zone 2 TEVAR with near complete resolution for PAU and TAI without the need for aortic reintervention.

Another topic of ongoing debate is the revascularization of the left subclavian artery, the coverage of which in patients undergoing TEVAR involves substantial risk of ischemic complications in the upper limb and central nervous system. Teixeira et al. found that revascularization of the left subclavian artery significantly reduced the risk of spinal cord ischemia [13]. Moreover, Zamor et al. showed that coverage of the left subclavian artery without revascularization significantly increased the risk of stroke and upper arm ischemia [14]. However, despite numerous reports of the benefit of left subclavian artery revascularization, the local surgical complications should be clarified together. We reported early complications related to the left subclavian artery bypass procedure including nerve injury in several patients. However, all of these local surgical complications improved during follow-up. Furthermore, these complications are considered to be minor in comparison to the possible adverse effects of subclavian artery coverage without revascularization. Neither left-arm claudication nor spinal cord ischemia were observed in our study, because the left subclavian artery revascularization procedure was successful. Therefore, our study supports the efficacy of zone 2 hybrid TEVAR with concomitant left subclavian artery bypass for the treatment of thoracic aortic diseases involving the left subclavian artery. Since we routinely performed left subclavian artery embolization simultaneously, the risk of type II endoleak was virtually eliminated.

TEVAR in general has been demonstrated to be safe, but the possibility of subsequent reintervention with endoleak is a well-known risk. Our study observed five cases of endoleaks (10.4%) with two cases of secondary reinterventions (13.3%) for increasing AAs due to endoleak. This result is consistent with those of previous studies, which revealed an incidence of endoleaks of 5–20% [15, 16]. All patients who underwent aortic reintervention for persistent endoleaks were initially diagnosed with AA with a maximal diameter of > 60 mm. Furthermore, 50% of the patients with large AA (diameter > 60 mm) showed persistent aneurysmal enlargement during the follow-up period. Endoleaks were significantly more common in patients with AA than in those with other aortic pathologies. In open surgical repair, the aneurysmal sac is completely removed, but in the case of TEVAR, only exclusions through stent-graft are allowed. The fate and impact of the residual aneurysm sac after TEVAR remains poorly defined. One study demonstrated that several preoperative morphological factors significantly affected the probability of sac expansion after TEVAR for thoracic aortic aneurysm [17]. The known predictors of endoleak include the anatomy of the arch, the lengths of the stent grafts covering the aorta, the diameter of the AA, the length of the proximal landing zone, and zone types [18–20]. Our data support that patients with large AAs are at a higher risk of endoleak and sac expansion. The strategy and selection of zone 2 hybrid TEVAR should thus potentially be based on the aortic pathology rather than only as an alternative to open repair for high-risk patients.

There are several limitations to this study. First, this was a retrospective observational study in which the data were gathered from review of the medical records. Second, all patients were enrolled from a single center and the number of patients was relatively small. Third, the statistical analysis was not performed to draw general conclusions. However, the follow-up data were complete in 100% of the patients, and all patients had annual CT scan records to compare the mid-term aortic measurements. Further studies should include larger samples to further validate the findings of our study.

## Conclusion

One-stage zone 2 hybrid TEVAR achieved excellent results in terms of early outcomes and mid-term aortic measurements during the follow-up period. The local complication rates during the early postoperative period had a limited impact on the late course and finally resolved without sequelae. However, in cases in which the initial diameter of the AA is > 60 mm, a considerable number of patients experienced confirmed postoperative persistent aneurysm enlargement with endoleak. Therefore, open surgical repair may be more suitable than zone 2 TEVAR for patients with large AAs.

## Data Availability

The datasets used and/or analysed during the current study are available from the corresponding author on reasonable request.
